# The Norwegian guidelines for the prehospital management of adult trauma patients with potential spinal injury

**DOI:** 10.1186/s13049-016-0345-x

**Published:** 2017-01-05

**Authors:** Daniel K Kornhall, Jørgen Joakim Jørgensen, Tor Brommeland, Per Kristian Hyldmo, Helge Asbjørnsen, Thomas Dolven, Thomas Hansen, Elisabeth Jeppesen

**Affiliations:** 1 East Anglian Air Ambulance, Cambridge, UK; 2 Department of Acute Medicine, Nordland Central Hospital, Postboks 1480, 8092 Bodø, Norway; 3 Swedish Air Ambulance, Mora, Sweden; 4 Department of Traumatology, Oslo University Hospital, Oslo, Norway; 5 Department of Vascular Surgery, Oslo University Hospital, Oslo, Norway; 6 Neurosurgical Department, Oslo University Hospital, Oslo, Norway; 7 Trauma Unit, Sørlandet Hospital, Kristiansand, Norway; 8 Department of Research, Norwegian Air Ambulance Foundation, Drøbak, Norway; 9 Department of Anesthesia and Intensive Care, Haukeland University Hospital, Bergen, Norway; 10 Helicopter Emergency Medical Services, Bergen, Norway; 11 Emergency Medical Services, University Hospital of North Norway, Tromsø, Norway; 12 Norwegian National Advisory Unit on Trauma, Oslo University Hospital, Oslo, Norway; 13 Department of Health Studies, University of Stavanger, Stavanger, Norway

**Keywords:** Prehospital emergency care, Spinal cord injury, Stabilisation, Airway management, Guideline

## Abstract

The traditional prehospital management of trauma victims with potential spinal injury has become increasingly questioned as authors and clinicians have raised concerns about over-triage and harm. In order to address these concerns, the Norwegian National Competence Service for Traumatology commissioned a faculty to provide a national guideline for pre-hospital spinal stabilisation. This work is based on a systematic review of available literature and a standardised consensus process. The faculty recommends a selective approach to spinal stabilisation as well as the implementation of triaging tools based on clinical findings. A strategy of minimal handling should be observed.

**Electronic supplementary material:**

The online version of this article (doi:10.1186/s13049-016-0345-x) contains supplementary material, which is available to authorized users.

## Background

Traumatic injury to the spinal cord (SCI) or cauda equina is uncommon but may have devastating consequences [1, 2]. *Spinal instability* occurs when the integrity of the spinal column is compromised by fractures and/or joint dislocations so that it no longer can maintain it’s protective configuration under normal physiologic loading, predisposing to further injury [3, 4]. Since the 1960s, mishandling of the traumatised spine has been thought to cause neurological deterioration and field spinal stabilisation has been considered pivotal for preventing such secondary injury [5–15]. Through adding external supports to the victim’s body before extrication, treatment and transport to hospital, clinicians aim to reduce spinal movement and prevent further secondary injury [16–19]. The spine is to be stabilised in a neutral position. While this position is poorly defined and subject to controversy and individual variation, it is similar to the position one assumes when standing and looking ahead [20–24]. For decades, the dominant strategy has been to generously assume the presence of unstable spinal injury in all patients with a relevant mechanism of injury or clinical findings and then to stabilise using a combination of a rigid cervical collar, head-blocks, straps and a rigid stretcher system [25–32]. While numerous other devices exist, this combination is widely implemented [33–39].

Many authors have raised concerns over this strategy and have questioned its efficacy, over-triage, costs and potential harmful effects [40–45]. Consequently, several organisations and authors have promoted a more selective strategy [2, 44, 46]. This controversy has generated regional variations in stabilisation strategies within the Emergency Medical Services (EMS) [44]. In order to address these concerns from a national perspective, the Norwegian National Competence Service for Traumatology (NKT-T) in collaboration with The Norwegian Knowledge Centre for the Health Services (NOKC) commissioned a multi-disciplinary faculty to provide a national guideline designed to facilitate the prehospital management of adult trauma victims with potential spinal injury. The GRADE system (grading of recommendations, assessment, development and evaluation) has been combined with standards for clinical practice guidelines and best available evidence to improve pre-hospital management of adult patients with potential spinal injury.

## Methods

The multi-disciplinary faculty included members from all Norwegian health trusts representing the medical specialties of neurosurgery (1), trauma surgery (1), prehospital care (3), anesthesiology (1) and EMS (1), all with expert knowledge of trauma management. In addition, one methodologist directed the systematic evidence work, including evidence appraisal and synthesis. The standards for developing clinical practice guidelines using The Appraisal of Guidelines for Research and Evaluation (AGREE) tool were followed [47]. Key clinical questions were created in accordance with the PICO format (Population, Intervention, Comparison, Outcome) (Table 1). In December 2014, a research librarian performed a scoping search for existing international guidelines and systematic reviews [48–52].

**Table 1:** Overview of key clinical questions in the PICO format

Clinical question	P	I	C	O
Does routine use of spinal stabilisation prevent secondary neurological injury?	Trauma population	Spinal stabilisation	Stabilisation vs no stabilisation	Neurological morbidity
Are there alternative ways of stabilising the spinal column?	Trauma population	Spinal stabilisation	collar/MILS/stretcher/backboard	Neurological morbidity Pain/discomfort
Is there evidence of harmful side effects caused by stabilisation devices?	Trauma population	Spinal stabilisation	Stabilisation vs no stabilisation	Neurological morbidity Pain, discomfort, ulceration
Are there sub-groups of patients that in particular should not be stabilised?	Critical injuries Minor injuries	No spinal stabilisation	Stabilisation vs no stabilisation	Neurological morbidity & mortality
How should patients with potential spinal injury be evacuated and transported?	Trauma population	Extrication & transport	Stretcher, vacuum mattress, backboard	Neurological morbidity & mortality Pain, discomfort, ulceration

*PICO* Population, Intervention, Comparator, Outcome

In March 2015, a systematic literature search for primary studies was performed on core databases Medline, Embase The Cochrane Library and the Cochrane Central Register of Controlled Trials (CENTRAL). Medical Subject Headings (MeSH) search terms are listed in Additional file 1 that is available as a supplementary on-line material. Search was further limited to human studies published in English language.

Two reviewers independently screened titles and abstracts of all records identified in the searches for inclusion. Any discrepancy was resolved through discussion and consensus in the faculty. For completeness, additional records were identified by scanning reference lists and the authors contributing papers known to them. Full text records were critically appraised using the PRISMA checklist for systematic reviews, the CASP checklist for observational studies and the AGREE tool for guidelines [47, 53]. The quality of evidence and strength of recommendations were described using the GRADE tool. In line with the principles of the GRADE methodology, we downgraded the quality of evidence of an intervention for identified risks of bias (methodological quality), inconsistency, indirectness, imprecision or publication bias. Evidence was rated as one of four levels of quality (high, moderate, low and very low). When agreeing on strength of recommendations, three factors were considered and integrated in a group consensus process: benefits and harms, quality of evidence and the preferences of patients and clinicians. The strength of recommendations were graded as strong or conditional. A strong recommendation indicates that the benefits of an intervention far outweigh the harms (or vice versa). A conditional recommendation denotes uncertainty over the balance of benefits and harms. Finally, the faculty opted to use the term ‘good clinical practice’ in instances where a recommendation was considered obviously rational, but where the literature was found too heterogeneous for meta-analysis.

## Results

Six guidelines were identified in the scoping stage [2, 19, 46, 54–56]. One publication was of particularly high methodological quality. In 2013, A joint committee from The American Association of Neurological Surgeons (AANS) and the Congress of Neurological Surgeons (CNS) issued updated guidelines for the management of acute cervical spine and spinal cord injuries [46]. These comprehensive guidelines are based on systematic literature searches between 1966 and 2011 and was considered to be both relevant and complete by our faculty. Therefore, we limited our further searches to papers published after 2010 overlapping the AANS/CNS joint committees’ searches by 1 year. In their guideline, Theodore et al. relied on 109 records for their literature review of which one was excluded as it was found as a duplicate amongst our search results. Of the remaining 108, 93 were available in full text format for inclusion into our literature review. The 15 records that were not available were non-peer-reviewed magazine articles, meeting proceedings, local EMS protocols and chapters in textbooks no longer in print or otherwise not available.

Our core database search generated 9.441 abstracts and titles. After independent author review, 9372 were excluded by title or abstract for unrelated topics or for not being primary studies or systematic reviews. A total of 69 original papers were selected for full text reading of which 16 were considered eligible for inclusion into our literature base (Additional file 2). In addition, six systematic reviews were identified and included (Additional file 3) [42, 44, 45, 57–59].

During guideline preparation, another 16 original studies were identified from screening bibliographies and from the authors contributing articles known to them. In total, we identified 63 original studies and 6 systematic reviews that generated and supported 10 recommendations.

**Fig. 1:**
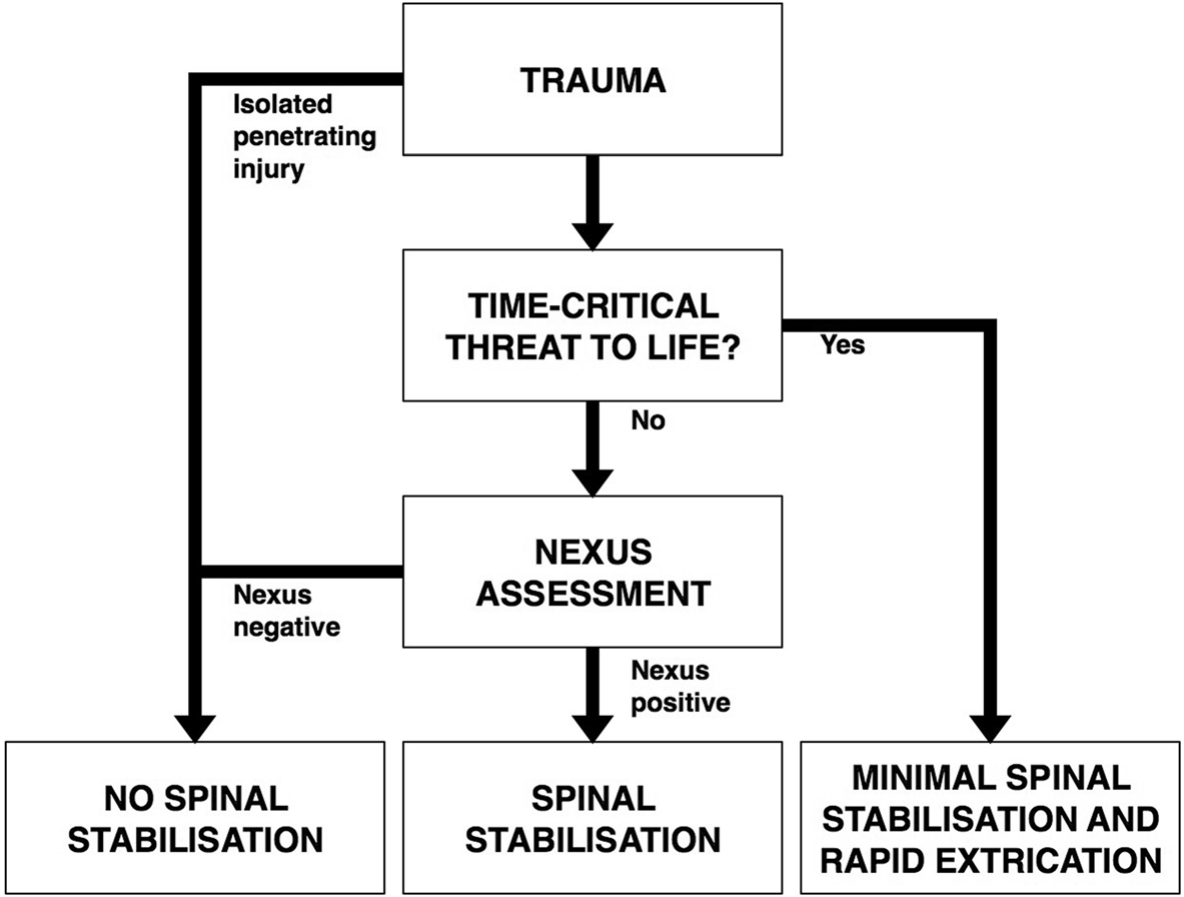
Flowchart describing pre-hospital spinal stabilisation in patients with suspected spinal injury

Our recommendations, the quality of supporting evidence as well as the strength of recommendation are summarised in Table 2. The original studies supporting each recommendation are listed and described in a separate evidentiary table that is available as supplementary material (Additional file 4). The recommendations formed the framework for an algorithm designed to facilitate the prehospital management of adult trauma victims with potential spinal injury (Fig. 1). A draft version of the guideline was subjected to a national open hearing process involving stakeholders such as the ambulance services of the Norwegian hospital trusts, the air ambulance organisations, regional trauma leaders and the primary health care services. This manuscript presents the finalised version of these recommendations. The rationale and literature behind each recommendation is expanded upon below.

**Table 2:** Summary of recommendations, quality of evidence and strength of recommendation

Recommendation		Quality of evidence	Strength of recommendation	Rationale (Benefits, harms and the preferences of patients and clinicians)
1	Victims with potential spinal injury should have spinal stabilisation.	Very low	Strong	Paucity of literature supporting spinal stabilisation. Very little literature documenting serious harm. Spinal cord injury can have devastating consequences. Potential benefits outweigh harms
2	A minimal handling strategy should be observed.	Very low	Strong	Paucity of literature supporting spinal stabilisation. Very little literature documenting serious harm. Spinal cord injury can have devastating consequences. Potential benefits outweigh possible harms
3	Spinal stabilisation should never delay or preclude life-saving intervention in the critically injured trauma victim.	Very low	Good clinical practice	Literature supporting this recommendation was considered too heterogenous for synthesis. The faculty finds that it is logical that spinal stabilisation in the critically injured patient may cause serious harm
4	Victims of isolated penetrating injury should not be immobilised.	Moderate	Strong	One large study of moderate quality directly supports this recommendation. Spinal injury in patients with isolated penetrating injury is rare
5	Triaging tools based on clinical findings should be implemented.	Moderate	Strong	Consistent evidence supporting triaging tools based on clinical findings rather than mechanism. No harmful effects documented
6	Cervical stabilisation may be achieved using manual in-line stabilisation, head-blocks, a rigid collar or combinations thereof.	Very low	Conditional	Consistent experimental evidence demonstrating how rigid collars can stabilise the cervical spine. However, there is also evidence suggesting harm from rigid collars. No evidence proving superiority of any one method
7	Transfer from the ground or between stretchers should be achieved using a scoop stretcher.	Very low	Conditional	General paucity of evidence. Some evidence for significant spinal motion during log-roll. Some evidence documenting improved stability with scoop stretcher transfers. Safety of scoop stretcher systems is good. No harmful effects documented
8	Patients with potential spinal injury should be transported strapped supine on a vacuum mattress or on an ambulance stretcher system.	Very low	Conditional	Evidence supporting harm from hard surface stretcher systems. No consistent evidence demonstrating increased stability with any one method. Increased comfort associated with soft surface systems. No evidence exploring spinal stability of common stretcher systems
9	Hard surface stretcher systems may be used for transports of shorter duration only.	Very low	Conditional	Evidence supporting harm from hard surface stretcher systems. No consistent evidence demonstrating increased stability with any one method. Increased comfort associated with soft surface systems
10	Patients should under some circumstances be invited to self-extricate from vehicles.	Very low	Conditional	Two experimental studies demonstrating improved stability with self-extrication from vehicles. Reasonable and practical alternative as long as used cautiously

The guideline is now undergoing national operationalisation as it is being implemented in the individual health trusts’ ambulance operating procedures, is taught on e-learning programs and is incorporated into the training of new EMS personnel. The guideline is also widely disseminated through meeting presentations and in national care publications

**Recommendation 1: Victims with potential spinal injury should have spinal stabilisation.**

**Recommendation 2: a minimal handling strategy should be observed.**

### Rationale and evidence base

#### Higher level evidence supporting spinal stabilisation is lacking

Despite spinal stabilisation being one of the most frequently performed prehospital interventions, higher grade evidence demonstrating beneficial effects is lacking [46]. From the 1970s to the 1980s the incidence and mortality of complete spinal injury decreased significantly. As this coincided with the introduction of modern spinal management strategies, authors have to varying extents credited stabilisation for this reduction [46, 60–63]. Other than these assumptions, the evidence directly supporting stabilisation consists of reports of low quality associating failure to reduce spinal mobility with neurological deterioration [6, 9, 64–67]. In contrast, in a controversial study comparing patients who had spinal stabilisation in New Mexico, USA with patients in Kuala Lumpur, Malaysia who had no spinal stabilisation, Hauswald et al. demonstrated no protective effect from stabilisation [68]. Nevertheless, taking into account the existing evidence, the anatomical perspective as well as decades of clinical experience, it is likely that the current paradigm of spinal stabilisation has played a part in the reduction of secondary neurological injury. The faculty found no reason to abandon the strategy of external spinal stabilisation. For the same reasons, after having restored the patient to an anatomical position, it is recommended that unnecessary patient handling and movement is minimised. Authors have opined that working in accordance with such a *minimal handling strategy* may not only reduce spinal movement but may also minimise pain as well as promote hemostasis [69].

**Recommendation 3: Spinal stabilisation should never delay or preclude life-saving intervention in the critically injured trauma victim.**

### Rationale and evidence base

While the faculty recommends adhering to the prehospital stabilisation doctrine, it must also be recognised that SCI is uncommon and that spinal stabilisation is not, in itself, always a benign intervention [43, 46].

#### Spinal stabilisation may interfere with or delay life-saving intervention

The incidence of SCI in hospitalised trauma victims has been reported in the range of 0.5 to 3% [2]. Spinal stabilisation may preclude or delay the effective management of life-threatening reversible insults such as airway compromise, hypoxemia, tension pneumothorax, cardiac tamponade, haemorrhage or brain trauma which may require urgent prehospital or hospital interventions. Spinal stabilisation has been associated with difficult airway management, restricted thoracopulmonary function and delayed time to intervention [42, 44, 70–74]. In light of this, spinal stabilisation must be de-emphasised in the critically injured patient. While remaining important, spinal stabilisation should never interfere with or delay life-saving intervention nor be allowed to cause or worsen critical injury (Fig. 1).

#### Recognising time critical injury

Staging and defining time critical injury in trauma is controversial. Identifying patients with critical injury that are unlikely to tolerate prolonged extrication and spinal stabilisation is ultimately up to the attending clinician. Vital parameters may support the decision but should be interpreted cautiously. Nevertheless, we have opted to include a supporting definition of critical injury previously issued by The Norwegian Directorate of Health in a guideline for the management of mass casualty incidents. This definition is designed for individual triage, is based on readily obtainable clinical findings and is nationally recognised by our EMS [75].

Airway compromise, respiratory rate lower than 10 or above 30 breaths per minute, pulse frequency above 120 beats per minute, absent radial pulses or no motor response to verbal commands are signs suggestive of time critical injury. Patients with injuries designated as time critical should not suffer prolonged extrication or evacuation due to spinal concerns. Importantly, this does not imply that spinal precautions are entirely abandoned, but only applied to an extent and in a way that does not delay extrication nor intervention.

#### The lateral trauma position

Historically, first responders without advanced airway skills have placed unconscious or obtunded victims in the recovery position in order to facilitate the clearance of fluids and to maintain airway patency [45]. Unfortunately, this contradicts the principle of spinal stabilisation in trauma victims as it generates unacceptable spinal movement [76]. The lateral trauma position (LTP) is a variation of the established recovery position that is achieved using a modified two-person log-roll with manual cervical spine control and, eventually, blankets and a rigid collar for cervical stabilisation [77]. While the log-roll involved may generate spinal motion, this may be offset by the beneficial effects of gaining airway patency and clearance. Clinicians not trained in advanced airway management should be encouraged to consider the LTP when transporting obtunded patients.

**Recommendation 4: Victims of isolated penetrating injury should not be immobilised.**

### Rationale and evidence base

Victims of isolated penetrating trauma suffer increased mortality with routine spinal stabilisation [57]. In a 2010 retrospective review of hospitalised victims of penetrating trauma, Haut et al. demonstrated how patients with penetrating injuries who had spinal stabilisation had twice the mortality (14.7%) as those who were not stabilised, likely through delaying transport to surgical intervention. Moreover, the authors found that spinal cord injury in isolated penetrating injury was extremely rare at a rate of 0.01% of victims [74].

**Recommendation 5: Triaging tools based on clinical findings should be implemented.**

### Rationale and evidence base

#### Triaging tools

In order to address over-triage, authors have advocated implementing triaging tools to assist in identifying low-risk patients who do not require stabilisation [41, 78–81]. The National Emergency X-radiography Utilisation Study (NEXUS) tool and the Canadian C-Spine Rule Criteria (CCR) were originally developed to aid physicians in determining which trauma patients require imaging of the cervical spine [2, 82, 83]. Protocols similar to NEXUS have proven to be useful triaging tools for prehospital spinal stabilisation. Since the early 1990s the Fresno/Kings/Madera EMS system in California have implemented a selective stabilisation protocol similar to NEXUS. In a 2001 retrospective review, Stroh and Braude reported that this protocol had a 99% sensitivity for the correct stabilisation of patients with actual cervical injury [84]. In a prospective observational study of EMS personnel in Maine, also using a tool similar to NEXUS, Burton et al. found that the protocol sensitivity for stabilisation of any spinal fracture was 87% with a negative predictive value of 99.9% [85].

#### Triaging tools based on clinical findings reduce over-triage

Authors have recommended implementing tools that, similar to NEXUS, are predominantly based on clinical findings [78, 86, 87]. Tools that emphasise the mechanism of injury result in over-triage without increasing accuracy. In a prospective review of 498 trauma patients, Hong et al. found that 95.4% of patients would have been immobilised if EMS personnel had stabilised in accordance with the mechanism based 7th edition PHTLS criteria. In contrast, stabilisation in accordance with protocols based on clinical findings, NEXUS or Hankins protocols, would result in stabilisation rates of 68.7% and 81.5%, respectively. All patients with actual spinal injury would have been stabilised using any of the protocols [88]. In 1999, Muhr et al. reported how the implementation of an out-of-hospital clearance protocol based on clinical findings reduced stabilisation by one third [87]. These and other reports provide evidence of how EMS successfully can implement selective prehospital stabilisation strategies given that they are coupled with training and clinical governance [89–93]. The faculty recommends applying the NEXUS criteria on the entire spine for triage in the prehospital setting. In the absence of midline tenderness, focal neurologic deficit, altered level of consciousness, intoxication, and significant distracting injury, it is safe to withhold stabilisation.

**Recommendation 6: Cervical stabilisation may be achieved using manual in-line stabilisation, head-blocks, a rigid collar or combinations thereof.**

### Rationale and evidence base

The approach to cervical stabilisation should be informed and selective, observing the pros and cons of several techniques. The goal is to achieve stabilisation of the cervical spine. The means will vary.

#### The efficacy and harms of the rigid cervical collar

No high quality studies have identified the true efficacy of the rigid collar. The existing evidence is difficult to compare due to variations in methodology and types of collars tested [94]. However, numerous studies document how the application of a rigid cervical collar will limit motion in the cervical spine [34, 36, 95–101]. It is also apparent, from these same studies, that movement restriction is limited. Moreover, there is a growing body of evidence documenting harm. As rigid collars achieve cervical stabilisation through compression of the mandible, mouth opening will be reduced. Thus, application may impede breathing and airway management including the clearing of vomit or secretions [43, 102]. Rigid cervical collars can increase intracranial pressure by inducing pain or through blocking cranial venous return [103–105]. In a study on cadavers with an artificially induced unstable C1-C2 lesion, Ben-Galim et al. demonstrated how cervical traction from a collar caused separation between C1 and C2, suggesting a mechanism that could aggravate injury [106]. Severe neurological deterioration has been reported in patients with ankylosing spondylitis after receiving triple stabilisation [107, 108]. Finally, rigid collars may induce pain or discomfort that may trigger non-compliance, agitation and even increased spinal movement in some patients [109–111]. Cervical collar use has also been associated with pressure point ulceration, necrosis and mandibular nerve palsy with prolonged use [112–116].

#### The rigid collar should not be applied routinely

The aforementioned reports support a selective approach to rigid collar use. While collars are safe to use in the majority of patients, they should be used selectively in patients with traumatic brain injury, airway compromise, ankylosing spondylitis or agitation. In such cases the collar may be withheld or used intermittently. The collar may provide support during certain manoeuvres, such as in stretcher transfers or during evacuation from a vehicle, after which the collar may be opened or removed [99, 117]. With adequate MILS this can be achieved with minimal spinal displacement [118]. Transport may proceed using only MILS and/or head blocks. Holla et al. recently demonstrated how the addition of a rigid collar did not result in improved movement restriction in volunteers already strapped to a rigid stretcher with head blocks [102]. Patients with a kyphotic spine, such as in ankylosing spondylitis, should be stabilised in a position similar to their habitual spinal curvature [108].

**Recommendation 7: Transfer from the ground or between stretchers systems should be achieved using a scoop stretcher.**

### Rationale and evidence base

A significant amount of spinal motion is generated as the patient is transferred from the ground onto or between stretcher systems or beds. Working in accordance with a minimal handling strategy, clinicians must take care to minimise spinal movement during these critical stages of extrication.

#### The log-roll may generate undue spinal motion and should be avoided in favour of alternative techniques

*Log-rolling* has traditionally been used to transfer the patient onto or off stretcher systems or to gain access to patients back for examination, despite authors questioning its safety [119]. The log-roll is a potentially dangerous procedure as it may cause fracture dislocation, pain, distress or clot disruption in patients with pelvic fractures or other injuries. The diagnostic value is limited [69, 120, 121]. Moreover, as the head, hips and pelvis are of different diameters, spinal motion is inherent to the technique, and several studies have demonstrated how log-rolling generates more motion than readily available alternative techniques such as lift-and-slide or *scoop stretcher* techniques [122–128]. The faculty believes that the potential spinal motion generated by the log-roll may be further aggravated in the prehospital setting where it is commonly performed with limited personell and under difficult working conditions. Usage of the technique in the prehospital context should therefore be minimised, if not abolished.

For transfers from the ground or between stretcher systems, we recommend employing a scoop stretcher system. As the scoop stretcher is split vertically and then reassembled underneath the patient, transfer from the ground or between stretchers requires minimal or no rolling [129]. Stabilisation and comfort has been demonstrated to be comparable or better than that of the classic backboard [122, 127, 130].

**Recommendation 8: Patients with potential spinal injury should be transported strapped supine on a vacuum mattress or on an ambulance stretcher system.**

**Recommendation 9: Hard surface stretcher systems may be used for transports of shorter duration only.**

### Rationale and evidence base

We wish to differentiate between hard and soft surface stretcher systems. Hard surface systems are those where the patient is directly lying on hard plastic or metal while soft surface systems have padding designed to increase comfort and decrease point pressure.

#### Hard surface stretcher systems

The *backboard* was designed to facilitate extrication but has since its inception been used as a transportation device and quickly became the gold standard for spinal stabilisation during transport [131, 132]. The literature, on the contrary, suggests that it is not appropriate for transports of longer duration. Within short time, patients will develop significant discomfort and moderate to severe pain [133–135]. Prolonged exposure may result in pressure ulcers [136, 137]. Pain and discomfort may also result in undue voluntary spinal movement [133]. The *scoop stretcher*, like the backboard, has hard surfaces that could induce pain, discomfort or pressure point injury. While it is an excellent extrication device and an appropriate transportation device for short distances, for longer duration transport the scoop stretcher, like the backboard, should be removed after transferring the victim onto a vacuum splint mattress or onto a standard ambulance trolley.

#### Soft surface stretcher systems

The *vacuum mattress*, while not rigid enough for extrication, is a useful transportation device. As vacuum is applied, the mattress moulds to the patient’s contours, minimising point pressure, making it more comfortable, less painful and, arguably, less likely to produce ulceration [73, 138–141]. The vacuum mattress has been shown to provide a similar, or superior, degree of stabilisation when compared to that of the backboard [132, 139, 140, 142].

**Recommendation 10: Patients should under some circumstances be invited to self-extricate from vehicles.**

### Rationale and evidence base

The traditional approach to extrication of victims with potential spine injury from vehicles or other settings has been to stabilise the victim with a cervical collar and then to carefully transfer the passive victim onto a backboard for extrication [143].

#### Self-extrication

Over the years, authors have argued that this practice often is unnecessary, resulting in prolonged extrication times and avoidable complications related to spinal stabilisation. Authors have argued that spinal movement within the normal range of motion requires so little energy, of many magnitudes less than the energy at the initial impact, that it is highly unlikely to cause further injury. Furthermore, the alert victim’s own muscular tone will suffice to protect the spine from further injury [41, 68, 144]. In 2013, the British Faculty of Pre-Hospital care acknowledged this in a statement recommending that the fully alert patient a potential spinal injury who is without distracting injury, should be allowed to self-extricate without external stabilisation [2]. Unfortunately, such a position is supported by very few studies. Shafer and Naunheim, in 2009, demonstrated how volunteers stabilised only with a rigid collar who exited a vehicle on their own volition, generated less spinal motion than when extricated using traditional assisted longboard techniques [145]. More recently, Dixon et al., in a biomechanical study on healthy volunteers found that controlled self-extrication without collar generated less movement in the cervical spine when compared to equipment aided extrication techniques [146].

#### A generous approach to self-extrication

Despite the scant evidence, we recommend self-extrication in some circumstances. As long as patients with back or neck pain are not obtunded, not under the influence of any drug, and without significant distracting injury, they should be invited to self-extricate to a nearby stretcher system. The prerequisite for self-extrication is that it is done under safe conditions. Should there be concerns about safety, then strategy defaults back to traditional extrication techniques. The patients should, after lying down on a stretcher system, have full external stabilisation for final evacuation and transport as they may then be subject to external force that may overwhelm their muscular protection.

## Summary

This guideline, based on consensus and the best available evidence, is an attempt to address concerns about over-triage, harms and costs associated with the traditional management of potential spinal injury. The faculty found no reason to abandon the current doctrine of spinal immobilisation in patients with potential spinal injury. We do, however, recommend implementing pre-hospital triaging tools as well as maintaining a selective approach to the use of the various stabilisation devices.

## Additional files

Additional file 1: Search engine vocabulary. (DOCX 108 kb)
Additional file 2: Table S1. Original studies identified in our search for new literature. (DOCX 24 kb)
Additional file 3: Table S2. The systematic reviews that were identified in our search for new literature. (DOCX 23 kb)
Additional file 4: Table S3. Original studies supporting our 10 recommendations. (DOCX 44 kb)

## Abbreviations

EMS: Emergency medical services
MILS: (Manual In-Line Stabilisation)
PHTLS: Pre-Hospital Trauma Life Support
SCI: Spinal Cord Injury
