# Supplementary material for: The Norwegian guidelines for the prehospital management of adult trauma patients with potential spinal injury
Source: Scand J Trauma Resusc Emerg Med. 2017 Jan 5;25:2. doi: 10.1186/s13049-016-0345-x (PMC5217292; doi:10.1186/s13049-016-0345-x)
Supplement: Additional file 1: — Search engine vocabulary. (DOCX 108 kb) [file 13049_2016_345_MOESM1_ESM.docx]

| **SEARCH ENGINE VOCABULARY** |
| --- |
| 16/12/14 The Cochrane Library |
| ID Search Hits  #1 (Spinal or spine or myelopath* or neck or cervix or cervical or brain or back or Whiplash or vertebra* or axis or dens or epistropheus or odontoid or atlant* or atlas or compressi*) near/2 (injur* or trauma* or fracture* or wound* or Polytrauma or transection* or laceration* or Post-Traumatic or "Post Traumatic" or contusion* or compressi* or dislocat* or displacement* or subluxation* or luxation*) or (Conus next Medullaris next Syndrome*):ti,ab,kw (Word variations have been searched) 10120  #2 MeSH descriptor: [Back Injuries] explode all trees 766  #3 MeSH descriptor: [Spinal Injuries] explode all trees 714  #4 MeSH descriptor: [Spinal Fractures] explode all trees 630  #5 MeSH descriptor: [Multiple Trauma] explode all trees 190  #6 MeSH descriptor: [Neck Injuries] explode all trees 204  #7 MeSH descriptor: [Whiplash Injuries] explode all trees 173  #8 MeSH descriptor: [Spinal Cord Injuries] explode all trees 902  #9 MeSH descriptor: [Central Cord Syndrome] explode all trees 0  #10 MeSH descriptor: [Spinal Cord Compression] explode all trees 107  #11 MeSH descriptor: [Trauma, Nervous System] this term only 18  #12 MeSH descriptor: [Spinal Cord Compression] explode all trees 107  #13 #2 or #3 or #4 or #5 or #6 or #7 or #8 or #9 or #10 or #11 or #12 2052  #14 #1 or #13 10307  #15 (Immobili* or precaution* or stabili* or stable or collar* or board* or backboard* or "back board*" or back-board* or extrication or hypokine* or orthotic or orthos* or brace* or restraint* or "vacuum mattress*" or splint* or "sand bag*" or MILS or " manual in-line stabilization" or "manual in-line stabilisation" or tape* or taping or strap* or packag* or stretcher* or litter* or trolley* or gurney* or BNDR or fixation):ti,ab,kw (Word variations have been searched) 46488  #16 MeSH descriptor: [Immobilization] explode all trees 625  #17 MeSH descriptor: [Restraint, Physical] explode all trees 221  #18 MeSH descriptor: [Braces] explode all trees 329  #19 MeSH descriptor: [Splints] explode all trees 380  #20 MeSH descriptor: [Stretchers] explode all trees 3  #21 #16 or #17 or #18 or #19 or #20 1269  #22 (Stif-neck or stiff-neck or "Stiff Neck" or x-collar or scoop or Kendrick or combicarrier* or skidboard or LBB or SAM or perfit or ACE og Redi-ACE*) (Word variations have been searched) 931  #23 #15 or #21 or #22 47343  #24 #14 and #23 1337 |

| [Ovid MEDLINE](http://proxy.helsebiblioteket.no/login?url=http://ovidsp.ovid.com/ovidweb.cgi?T=JS&NEWS=n&CSC=Y&PAGE=main&D=pmez) og [Embase](http://proxy.helsebiblioteket.no/login?url=http://ovidsp.ovid.com/ovidweb.cgi?T=JS&MODE=ovid&NEWS=n&PAGE=main&D=emez) ( 2010-) | |
| --- | --- |
| 17.03.2015 | |
| 1 exp Back Injuries/  2 exp Spinal Injuries/  3 exp Spinal Fractures/  4 exp Multiple Trauma/  5 exp Neck Injuries/  6 exp Whiplash Injuries/  7 exp Spinal Cord Injuries/  8 exp Central Cord Syndrome/  9 exp Spinal Cord Compression/  10 Trauma, Nervous System/  11 (((Spinal or spine or myelopath* or neck or cervix or cervical or brain or back or Whiplash or vertebra* or axis or dens or epistropheus or odontoid or atlant* or atlas or compressi*) adj2 (injur* or trauma* or fracture* or wound* or Polytrauma or transection* or laceration* or Post-Traumatic or "Post Traumatic" or contusion* or compressi* or dislocat* or displacement* or subluxation* or luxation*)) or (Conus adj Medullaris adj Syndrome*)).tw.  12 or/1-11  13 exp Orthotic Devices/  14 exp Braces/  15 exp Splints/  16 exp Stretchers/  17 ((vacuum adj4 splint*) or Immobili* or precaution* or stabili* or stable or collar* or board* or backboard* or "back board*" or extrication or orthotic or orthos* or brace* or restraint* or "vacuum mattress*" or splint* or "sand bag*" or MILS or "manual in-line stabilization" or "manual in-line stabilisation" or tape* or taping or strap* or packag* or stretcher* or gurney* or "head block*" or headblock* or fixation).tw.  18 or/13-17  19 12 and 18  20 19 use pmfc  21 exp spine injury/  22 exp cervical spine injury/  23 exp spine fracture/  24 exp odontoid process fracture/  25 exp vertebra compression/  26 exp vertebra dislocation/  27 exp atlantoaxial dislocation/  28 exp atlantoaxial subluxation/  29 exp cervical spine dislocation/  30 (((Spinal or spine or myelopath* or neck or cervix or cervical or brain or back or Whiplash or vertebra* or axis or dens or epistropheus or odontoid or atlant* or atlas or compressi*) adj2 (injur* or trauma* or fracture* or wound* or Polytrauma or transection* or laceration* or "Post Traumatic" or contusion* or compressi* or dislocat* or displacement* or subluxation* or luxation*)) or (Conus adj Medullaris adj Syndrome*)).tw.  31 or/21-30  32 exp immobilization/  33 exp cervical collar/  34 exp rigid collar/  35 exp spine board/  36 exp spine stabilization/  37 exp splint/  38 exp stretcher/  39 ((vacuum adj4 splint*) or Immobili* or precaution* or stabili* or stable or collar* or board* or backboard* or "back board*" or extrication or orthotic or orthos* or brace* or restraint* or "vacuum mattress*" or splint* or "sand bag*" or MILS or "manual in-line stabilisation" or tape* or taping or strap* or packag* or stretcher* or gurney* or "head block*" or headblock* or fixation).tw.  40 or/32-39  41 31 and 40  42 41 use emefd  43 20 or 42  44 limit 43 to yr="2010"  45 remove duplicates from 44  46 limit 43 to yr="2011"  47 remove duplicates from 46  48 limit 43 to yr="2012"  49 remove duplicates from 48  50 limit 43 to yr="2013"  51 remove duplicates from 50  52 limit 43 to yr="2014"  53 remove duplicates from 52  54 limit 43 to yr="2015"  55 remove duplicates from 54  56 or/45,47,49,51,53,55  57 limit 56 to (danish or english or german or norwegian or swedish)  58 limit 57 to (addresses or autobiography or bibliography or biography or clinical conference or comment or congresses or consensus development conference or consensus development conference, nih or "corrected and republished article" or dataset or dictionary or directory or duplicate publication or editorial or evaluation studies or festschrift or historical article or in vitro or interactive tutorial or interview or lectures or legal cases or legislation or letter or news or newspaper article or patient education handout or periodical index or portraits or twin study or validation studies or video-audio media or webcasts or book or book series or conference abstract or conference paper or conference proceeding or "conference review" or note or report or short survey or trade journal) [Limit not valid in Ovid MEDLINE(R),Ovid MEDLINE(R) In-Process,Embase; records were retained]  59 57 not 58 | |
| **Antall treff** | 9441 |
| **Kommentarer** | I Ovid er det ikke er mulig å «dedupe» (fjerne dubletter) fra sett på mer enn 6000 treff, derfor ble denne prosessen delt opp ved å først begrense på år og så «dedupe» (linje 44-55). I linje 56 ble treffene fra de «dedupede» linjene samlet. På grunn av enorm treffmengde, valgte gruppen å begrense til publikasjoner fra og med 2010. Ettersom treffmengden var svært stor (ca 14000 treff) selv etter begrensning på år, valgte vi også å begrense på språk og publikasjonstyper. De 9441 treffene ble eksportert til EndNote. |
