# Supplementary material for: The Norwegian guidelines for the prehospital management of adult trauma patients with potential spinal injury
Source: Scand J Trauma Resusc Emerg Med. 2017 Jan 5;25:2. doi: 10.1186/s13049-016-0345-x (PMC5217292; doi:10.1186/s13049-016-0345-x)
Supplement: Additional file 2: Table S1. — Original studies identified in our search for new literature. (DOCX 24 kb) [file 13049_2016_345_MOESM2_ESM.docx]

| **First author, Year** | **Study Design** | **N** | **Intervention** | **Results** | **Study Quality (CASP)** |
| --- | --- | --- | --- | --- | --- |
| Horodyski, 2011 | Experimental | 5 cadavers | Range of cervical motion (ROM) | No significant difference with vs no collar | Low |
| Holla, 2010 | Experimental | 10 healthy volunteers | ROM with rigid cervical collar, head blocks, both | Reduced ROM with cervical collar (34%) or head blocks (12%). No benefit from using both | Low |
| Ivanic, 2013 | Experimental | 8 specimens | Flexibility tested with four different collars | Significantly reduced ROM for all collars | Low |
| Lin, 2011 | Retrospective cohort | 5,139 lightweight motorcycle injuries | Pre-hospital cervical collar vs no collar | 50.7% with collar. 63 patients with cervical spine injuries. 74.6% neurological deficiency. No significant difference | Moderate |
| Mahshidfar, 2013 | RCT | 60 trauma patients | Long backboard vs Vacuum mattress splint | LBB was faster, more effective, easier and more comfortable | Low |
| Mok, 2013 | Case-control | 60 military casualties vs 30 historic controls | 60 patients transported in VSB compared to 30 controls (non-VSB) | No cases of progressive neurological deficit or deformity, significant  higher incidence of pressure ulcers in intubated patients | Moderate |
| Morrison, 2014 | Retrospective cohort | 300 blunt trauma patients | Recorded NEXUS criteria and indication for imaging NEXUS-negative patients | 128 NEXUS positive, 91% imaging, 5 positive findings. 172 NEXUS negative, 30% underwent imaging, 2 positive findings | Moderate |
| Prasarn, 2012 | Experimental | 5 cadavers | One- or two-piece collars, instability (C5/C6) | Significant difference between the one-piece and two-piece collars | Low |
| Ben-Galim, 2010 | Experimental | 9 cadavers | Extrication collar applied, CT scans thereafter (C1/C2) | Extrication collar may cause worsening C1-C2 injury | Low |
| Boland, 2014 | Retrospective cohort | 1,394 patients with femur fractures | Determines the risk of c-spine injury with femur fractures | 1.7% of 1,394 had a c-spine injury | Moderate |
| Bruijns, 2013 | Cohort | 53 trauma patients | Effect of spinal immobilization on vital parameters | Spinal immobilization does not affect HR, BP and RR in healthy subjects | Low |
| Conrad, 2010 | Experimental | 3 cadavers | Testing hospital bed-transfer techniques (C5/C6) | No statistical difference | Low |
| Del-Rossi, 2013 | Experimental | 5 cadavers | Search for optimal alignment of the unstable spine | No difference between pads | Low |
| Stevens, 2015 | Retrospective cohort | 135 trauma patients | Self-extrication from car wreck | None of the spinal injuries (n=9) were unstable or led to surgery or neurological deficit | Low |
| Tello, 2014 | Retrospective cohort | 101 trauma patients | Immobilization by ED personnel | None of the patients secondarily immobilized had any missed acute cervical injury | Low |
| Haut, 2010 | Retrospective cohort | 45,284 patients with penetrating injury | Spinal immobilisation vs no immobilisation | Patients who underwent immobilisation were twice as likely to die (14.7% vs 7.2%). OR 2, 06, p<0.001 | High |
