# Supplementary material for: The Norwegian guidelines for the prehospital management of adult trauma patients with potential spinal injury
Source: Scand J Trauma Resusc Emerg Med. 2017 Jan 5;25:2. doi: 10.1186/s13049-016-0345-x (PMC5217292; doi:10.1186/s13049-016-0345-x)
Supplement: Additional file 3: Table S2. — The systematic reviews that were identified in our search for new literature. (DOCX 23 kb) [file 13049_2016_345_MOESM3_ESM.docx]

| **First author, Year** | **Search** | **Included studies** | **Recommendations** | **Methodological quality (PRISMA)** |
| --- | --- | --- | --- | --- |
| Oteir  2015 | October 2013 | Four observational studies | There is evidence in the literature that cervical spine and spinal immobilisation should be avoided when dealing with penetrating neck trauma. The practice of spinal immobilisation remains controversial in regards to its possible benefits, or harms, in blunt trauma patients. | High |
| Sundstrøm  2014 | April  2013 | 50 observational studies | Few patients are in need of spinal immobilization, and clearance protocols should be optimized to identify these high-risk patients. These patients should not be fitted with a collar, but immobilized on spine boards with head blocks and straps. Non-intubated trauma patients should be transported in a modified lateral recovery position that maintains near neutral spine alignment and airway patency. Prehospital management should, by no means, delay transportation of critically injured patients to definitive care. A safe, effective immobilization strategy is needed. | Moderate |
| Stuke  2011 | 2011 | 20 observational studies | There are no data to support routine spine immobilization in patients with penetrating trauma to the neck or torso or isolated penetrating trauma to cranium. Spine immobilization should never be done at the expense of accurate physical examination or identification and correction of life-threatening conditions in patients with penetrating trauma. Spinal immobilization may be performed after penetrating injury when a focal neurologic deficit is noted on physical examination although there is little evidence of benefit even in these cases. | Moderate |
| Ahn  2011 | April  2008 | 43 studies  (RCT, observational studies)  4 research questions. | Immobilization of patients with SCI during the prehospital setting should include a cervical collar, head immobilization, and a spinal board. Patients should be transferred off the hardboard on admission to a facility as soon as is feasible to minimize time on the hardboard. Airway management of acute SCI patients requiring intubation in the pre-hospital setting should include the use of manual in-line cervical spine traction. Intubation of patients with acute SCI in the pre-hospital setting should not rely solely on cervical collar neck immobilization. Indirect methods of intubation may cause less cervical movement than with direct laryngoscopy with a Miller blade. Emergency medical personnel in the pre-hospital setting can be trained to apply criteria to clear patients of cervical spinal injuries and immobilize patients suspected of having a cervical spinal injury. | Moderate |
| Kwan  2009 | July  2007 | No included studies | Spinal immobilisation (particularly of the cervical spine) can contribute to airway compromise, the possibility that immobilisation may increase mortality and morbidity cannot be excluded. Large prospective studies are needed to validate the decision criteria for spinal immobilisation in trauma patients with high risk of spinal injury. In addition, randomised controlled trials to compare different immobilisation strategies on trauma patients need to be considered in order to establish an evidence base for the practice of pre-hospital spinal immobilization. | High |
| Armstrong 2007 | 2007 | 105 audit forms | Ambulance personnel can carry out prehospital c-spine clearance safely and effectively. | Moderate |
