# Supplementary material for: The Norwegian guidelines for the prehospital management of adult trauma patients with potential spinal injury
Source: Scand J Trauma Resusc Emerg Med. 2017 Jan 5;25:2. doi: 10.1186/s13049-016-0345-x (PMC5217292; doi:10.1186/s13049-016-0345-x)
Supplement: Additional file 4: Table S3. — Original studies supporting our 10 recommendations. (DOCX 44 kb) [file 13049_2016_345_MOESM4_ESM.docx]

| **First author, Journal, Year** | **Study Design** | **Study group N** | **Study purpose** | **Results** | **Quality of evidence (GRADE)** |
| --- | --- | --- | --- | --- | --- |
| **RECOMMENDATION 1: VICTIMS WITH POTENTIAL SPINAL INJURY SHOULD HAVE SPINAL STABILISATION.**  **RECOMMENDATION 2: A MINIMAL HANDLING STRATEGY SHOULD BE OBSERVED.** | | | | | |
| Marshall, J Neurosurg, 1987 | Prospective multicenter study | 283 patients with SCI | Identify patients who deteriorated during hospital management | In 12 of the 14 patients who deteriorated neurologically the event was associated with a management (spinal movement) | Very low |
| Todd, Bone Joint J, 2015 | Medicolegal retrospective analysis | 59 patients with SCI | Assess frequency and causes for neurological deterioration | Consensus opinion of authors: Of 27 patients with altered neurological status, 25 could have been avoided | Very low |
| Toscano, Paraplegia, 1988 | Singel center retrospective review | 123 patients with spinal injury | Assess frequency and causes for neurological deterioration | 32 patients suffered neurological deterioration from time of injury to hospitalisation | Very low |
| Hauswald, Acad Emerg Med, 1998 | Retrospective chart review comparing SCI-patients in New Mexico and Malaysia | 120 patients in Malaysia (no immobilisation)  334 in New Mexico (immobilised) | Spinal stabilisation versus no stabilisation | Neurological deterioration was more common in New Mexicothan in Malaysia | Very low |
| Geisler, Med Serv J Can, 1966 | Retrospective review | 958 patients with SCI 29 patients | Assess frequency and causes for neurological deterioration | 29 patients with delayed onset paraplegia. Each case a consequence of failure to recognise the injury and to protect the spine | Very low |
| **RECOMMENDATION 3: SPINAL STABILISATION SHOULD NEVER DELAY OR PRECLUDE LIFE-SAVING INTERVENTION IN THE CRITICALLY INJURED TRAUMA VICTIM.** | | | | | |
| Haut, J Trauma, 2010 | Retrospective cohort study | 45,284 patients with penetrating injury | Spinal immobilisation vs no immobilisation | Patients who underwent immobilisation were twice as likely to die (14.7% vs 7.2%). OR 2, 06, p<0.001 | Moderate |
| Totten, Prehospital Emerg Care, 1999 | Randomised cross-over study | 39 healthy volunteers | The effect of backboard vs vacuum mattress | Both method resulted in similar (15%) respiratory restriction. Vacuum mattress significantly more comfortable | Very low |
| Bauer, Ann Emerg Med, 1988 | Prospective study | 15 healthy volunteers | Backboard vs Zee Extrication device in regards to pulmonary function | Both devices cause a significantly restrictive effect on pulmonary function | Very low |
| Schafermeyer, Ann Emerg Med, 1991 | Prospective study | 51 healthy children | Two different strapping configurations. | Spinal immobilisation significantly reduced respiratory capacity using both strapping techniques | Very low |
| **RECOMMENDATION 4: VICTIMS OF ISOLATED PENETRATING INJURY SHOULD NOT BE IMMOBILISED.** | | | | | |
| Haut, J Trauma, 2010 | Retrospective cohort study | 45,284 patients with penetrating injury | Spinal immobilisation vs no immobilisation | Patients who underwent immobilisation were twice as likely to die (14.7% vs 7.2%). OR 2, 06, p<0.001 | Moderate |
| **RECOMMENDATION 5: TRIAGING TOOLS BASED ON CLINICAL FINDINGS SHOULD BE IMPLEMENTED.** | | | | | |
| Domeier, Prehosp Emerg Care, 1997 | Retrospective chart review | 331 patients with spinal fractures | Identify clinical markers associated with spinal injury | Good correlation of spinal injury with clinical markers | Low |
| Lin, Am J Emerg Med, 2011 | Retrospective review | 8,633 patients from light motorcycle crashes | Determine incidence of cervical spinal injury | 63 of 8,633 patents had cervical spine injury. Average ISS 14.3+-8.3 | Low |
| Boland, Prehos Disaster Med, 2014 | Retrospective review | 1,394 patients with low level falls and hip fracture | Determine incidence of cervical spinal injury | Incidence of cervical spine fracture in this population was 1.7% | Very low |
| Morrison, Am J Emerg Med, 2014 | Cohort study | 300 patients of which 169 received c-spine imaging | Determine variables that causes imageing in NEXUS negative patients | 96% NEXUS neg receiving c-spine imaging were >65yrs.  Imaging revealed 7 positiv findings, of which 2 were in NEXUS negative patients | Low |
| Hoffman, N Eng J Med, 2000 | Prospective multicenter study | 34,069 patients with potential c-spine injury | Create clinical decision instrument | NEXUS criterias have a sensitivity of 99.0%, specificity 12.9%. NPV 99.8%, PPV 2.7% in ruling out significant cervical injury | Moderate* |
| Stroh, Ann Emerg Med, 2001 | Retrospective, multicenter study | 504 patients with c-spine injury | Clinical decision instrument for spinal stabilisation | 99% of CSI were immobilised by EMS using clinical decision tool | Very low |
| Burton, J Trauma, 2006 | Retrospective multicenter study | 31,885 trauma patients transported by EMS personnel | Do EMS immobilisation decisions correlate with spine fractures? | More than half EMS trauma patients avoided spinal immobilisation | Moderate* |
| Domeier, Prehosp Emerg Care, 1999 | Multicenter prospective cohort | 6,500 patients with potential spinal injury | Does mechanism of injury predict SCI? | Mechanism of injury does not predict SCI | Low |
| Muhr, Prehosp Emerg, 1999 | Prospective trial cohort compared with historical cohort | 281 patients with potential spinal injury | EMS personnel using clinical decision tool | Reduced spinal immobilisation by a third | Very low |
| Hong, West J Emerg Med, 2014 | Cross sectional study | 498 patients with potential c-spine injury | Prospective trial comparing 3 protocols for c-spine clearance | Rates of immobilisation: PHTLS 95.4%, NEXUS 68.7%, Hankins 81.5%  18 CSI, all decision tools would have immobilised them. | Very low |
| **RECOMMENDATION 6: CERVICAL STABILISATION MAY BE ACHIEVED USING MANUAL IN-LINE STABILISATION, HEAD-BLOCKS, A RIGID COLLAR OR COMBINATIONS THEREOF.** | | | | | |
| Graziano, Ann Emerg Med, 1987 | Cohort study | 45 healthy volunteers | Study of stabilisation devices using radiographic measures | Pre-hospital cervical stabilisation devices all reduced spinal mobility | Very low |
| Chandler, Ann Emerg Med, 1992 | Cohort study | 21 healthy volunteers | Study of cervical mobility using collar and spine board | Cervical collar and spine board in combination superior to collar alone | Very low |
| Podolsky, J Trauma, 1983 | Cohort study | 25 healthy volunteers | Study of cervical range of motion using different stabilisation devices in 25 volunteers | A combination of sandbags, tape and collar give the best cervical stabilisation | Very low |
| McCabe, Ann Emerg Med, 1986 | Cohort study | 7 healthy volunteers | Ability of different collars to stabilise the cervical spine | Difference between various collars identified | Very low |
| McGuire, Spine, 1990 | Experimental study | 3 cadaver | Ability of different collars to stabilise the cervical spine | Difference between various collars identified | Very low |
| Rosen, Ann Emerg Med, 1992 | Cohort study | 15 healthy volunteers | Ability of different collars to stabilise the cervical spine | Difference between various collars identified | Very low |
| Del Rossi, Spine, 2004 | Experimental study | 5 cadavers | To evaluate the controlling effects of cervical collars in transfer techniques | Tested collars functionally identical | Very low |
| Horodyski, J Emerg Med, 2011 | Experimental study | 5 cadavers | Measure degree of cervical stabilisation using various collars | Collars better than no collar, but motion restriction limited | Very low |
| Ivancic, Spine, 2013 | Experimental | 5 cadavers | Determine effectiveness of cervical collars and cerviothoracic orthosis | Cervicothoracic orthosis more effective than collars | Very low |
| Raphael, Anaesthesia, 1994 | Cohort study | 9 patients without head injury | Determine lumbar CSF pressure measured w/wo a cervical collar | 7 of 9 patients had a significant increase in lumbar CSF pressure with the cervical collar on | Very low |
| Davies, Injury, 1996 | Cohort study | 19 patients with head injury | Injured patients with ICP measurement probe tested w/wo a cervical collar | Collars significantly increased ICP | Very low |
| Kolb, Am J Emerg Med, 1999 | Cohort study | 20 patients without head injury | Determine lumbar CSF pressure measured with and without a cervical collar | Though statistically higher values of lumbar CSF pressure was noted, the clinical relevance was considered uncertain | Very low |
| Ben-Galim, J Trauma, 2010 | Experimental study | 9 cadavers | Study effect of cervical collar on unstable C1/C2 injury | Application of collars resulted in abnormal distraction at the studied level | Very low |
| Thumbikat, Spine, 2007 | Retrospective review | 18 patients with SCI | 10-year material identifying all admitted patiens with SCI and ankylosing spondylitis | Extension of the ankylosed kyphotic spine resulted in secondary neurological deficits | Very low |
| Plaisier, J Trauma, 1994 | Cohort study | 20 healthy volunteers | Volunteers tested various collars with skin pressure monitoring in order to determine risk of skin ulceration | Some collars more prone to ulceration than others | Very low |
| Lernet, Prehosp Emerg Care, 1998 | Randomized cross-over study | 39 healthy volunteers | Investigate incidence and severity of pain in volunteers undergoing spinal stabilisation | Pain is very frequently reported when spinal stabilisation is performed | Very low |
| Brujins, Prehosp Disaster Med, 2013 | Cohort study | 53 healthy volunteers | Testing blood pressure, heart rate and respiratory rate in volunteers undergoing spinal immobilisation | Pain was frequently reported, but vital signs did not differ during spinal immobilisation | Very low |
| Blaylock, Ostomy Wound Manag, 1996 | Cohort study | 20 patients | Identification of risk factors for skin ulceration in patients with cervical collars | Identifying risk factors and adjusting for these resulted no skin ulceration in 20 patients | Very low |
| Black, J Trauma Nurs, 1998 | Cohort study | 20 healthy volunteers | Measurement of skin pressure, temperature og humidity using various collars in volunteers | No clinically signifiant differences among the collars | Very low |
| Prasarn, J Trauma Acute Care Surg, 2012 | Experimental study | 5 cadavers | Amount of angular motion tested in 5 cadavers using different collars | Difference in angular motion between one -and two piece collars noted | Very low |
| Holla, J Emerg Med, 2012 | Cohort study | 10 healthy volunteers | Volunteers test range of motion using either head block, collars or a combination | Adding cervical collar to head blocks unnecessary | Very low |
| **RECOMMENDATION 7: TRANSFER FROM THE GROUND OR BETWEEN STRETCHERS SHOULD IDEALLY BE PERFORMED USING A SCOOP STRETCHER SYSTEM** | | | | | |
| Suter, Prehospital and Disaster Medicine, 1992 | Cohort study | 14 healthy adults | 5 variations of the log-roll maneuvre | The log-roll generates substantial spinal motion. Log-roll with arms extended generated least motion | Very low |
| McGuire, J Trauma, 1987 | Experimental study | 3 cadavers | Radiographic measurement of spinal movement when log-rolling, placement on backboard and on scoop stretcher | The spine board and scoop stretcher offer acceptable stabilisation of the unstable thoracolumbar spine. Substantial displacements of unstable thoracolumbar segments with log-rolling | Very low |
| Conrad, Spine, 2007 | Experimental study | 3 cadavers | Log-roll vs kinetic treatment table (KTT) | Log-rolling caused significantly more spinal motion compared to rolling on KTT | Very low |
| Del Rossi, J Athl Train, 2008 | Experimental study | 5 cadavers | Log-roll vs lift-and-slide vs 6-person-plus lift when transferring pt to a spine board | The log-roll caused significantly more motion in the unstable spine | Very low |
| Horodyski, J Trauma, 2011 | Experimental study | 5 cadavers | Log-roll vs lift-and-slide when transferring from spine board. | Spine boards can be removed using a lift-and-slide maneuver with less motion than when using the log-roll. | Very low |
| Del Rossi, Am J Emerg Med, 2010 | Experimental study | 5 cadavers | Transfer onto stretcher using scoop stretcher, vs. log roll vs. lift and slide. | The log roll created insignificant more motion in all directions than either the lift-and-slide technique or with scoop stretcher. The scoop stretcher and lift-and-slide were able to restrict motion to a comparable degree. | Very low |
| Krell, Prehosp, Emerg Care, 2006 | Experimental study | 31 healthy volunteers | Scoop stretcher vs long backboard at baseline, application, log-roll and lifting | The scoop stretcher caused significantly less movement on application and was more comfortable | Very low |
| **Recommendation 8: Patients with potential spinal injury should be transported strapped supine on a vacuum mattress or on an ambulance stretcher system.**  **Recommendation 9: HARD SURFACE STRETCHER SYSTEMS MAY BE USED for transports of shorter duration ONLY.** | | | | | |
| Chan, Ann Emerg Med, 1994 | Cohort study | 21 healthy volunteers | The effect of spinal stabilisation with regard to pain and discomfort | 100% of patients developed pain. 55% graded their symptoms as moderate-severe | Very low |
| Cordell, Ann Emerg Med, 1995 | Prospective crossover study | 20 healthy volunteers | The effect of air mattress in addition to collar and spine board | Immobilisation without the air mattress generated significantly more pain | Very low |
| Walton, Acad Emerg Med, 1995 | Prospective crossover study | 30 healthy volunteers | Padded vs non-padded spine-board immobilisation for 30 minutes | Significantly less discomfort when closed-cell foam padding was used | Very low |
| Linares, Orthopedics, 1987 | Cohort study | 32 patients with SCI | The effect of immobilisation on pressure sore development | Patients that developed pressure sores had not been turned frequently in the immediate post-injury period | Very low |
| Mawson, Am J Phys Med Rehabil, 1988 | Cohort study | 39 patients wih SCI | The incidence of pressure ulcers in patients who were immobilised in the immediate postinjury period | Association between pressure sores and immobilisation in the immediate post-injury period | Very low |
| Hamilton, J Emerg Med, 1996 | Prospective crossover study | 26 healthy volunteers | Effect of immobilisation on backboard vs vacuum splint w/wo collar in terms of discomfort | A significant difference in comfort, favouring the vacuum splint, was found. For all movement planes, except extension, the vacuum mattress provided superior cervical immobilisation | Very low |
| Johnson, Am J Emerg Med, 1996 | Prospective comparative study | 30 healthy volunteers | Vacuum splint vs backboard with respect to comfort, speed of application and degree of immobilisation | Vacuum splints were significantly more comfortable and provide a similar degree of immobilisation and speed of application | Very low |
| Mok, Spine J, 2013 | Case-control | 60 military casualties vs 30 historic controls | 60 patients transported on vacuum spine board compared to 30 controls | No cases of progressive neurological deficit or deformity, significant higher incidence of pressure ulcers in intubated patients | Very low |
| Totten, Prehosp Emerg Care, 1999 | Randomised cross-over study | 39 healthy volunteers | The effect of backboard vs vacuum mattress on respiration | Both method resulted in similar (15%) respiratory restriction. Vacuum mattress significantly more comfortable | Very low |
| Chan, J Emerg Med, 1996 | Prospective crossover study | 37 healthy volunteers | Effect of backboard vs vacuum mattress splint with regard to discomfort | Backboard immobilisation was associated with an increased incidence of pain and discomfort in general and occipital and lumbosacral pain in particular | Very low |
| Luscombe , Emerg Med J, 2003 | Prospective study | 9 healthy volunteers | The effect of backboard vs vacuum mattress with regared to discomfort | In the measured planes the vacuum mattress provided superior stability and comfort | Very low |
| Mahshidfar, Prehosp Disast Med, 2013 | Randomised clinical trial | 60 trauma victims | Backboard vs vacuum mattress splint | Immobilisation using the backboard was easier, faster, more comfortable for the patient and provided additional decrease in spinal movement | Very low |
| **Recommendation 10: PATIENTS should under some circumstances be invited to self-extricate FROM VEHICLES.** | | | | | |
| Stevens, J Emerg Med, 2015 | Retrospective study | 135 trauma patients | Safety of self-extrication from car wreck | None of the spinal injuries (n=9) were unstable or led to surgery or neurological deficit | Very low |
| Shafer, Western J Emerg Med, 2009 | Cohort study | 1 volunteer extricated using four techniques | Self-extrication from automobile vs standard assisted extrication technique. With vs without cervical collar | Least movement of the cervical spine in subjects who self-extricated with a cervical collar | Very low |
| Dixon, Emerg Med J, 2015 | Prospective comparative study | 16 healthy volunteers | 6 extrication techniques including extrication under own volition | Self-extrication causes less movement of the cervical spine than extrications performed using traditional assisted techniques | Very low |
